# Stroke Lesion Impact on Lower Limb Function

**DOI:** 10.3389/fnhum.2021.592975

**Published:** 2021-02-01

**Authors:** Silvi Frenkel-Toledo, Shay Ofir-Geva, Lihi Mansano, Osnat Granot, Nachum Soroker

**Affiliations:** ^1^Department of Physical Therapy, Faculty of Health Sciences, Ariel University, Ariel, Israel; ^2^Department of Neurological Rehabilitation, Loewenstein Rehabilitation Medical Center, Ra’anana, Israel; ^3^Sackler Faculty of Medicine, Tel Aviv University, Tel Aviv, Israel

**Keywords:** stroke, lower extremity, brain mapping, restitution, compensation, impairment, activity limitation

## Abstract

The impact of stroke on motor functioning is analyzed at different levels. ‘Impairment’ denotes the loss of basic characteristics of voluntary movement. ‘Activity limitation’ denotes the loss of normal capacity for independent execution of daily activities. Recovery from impairment is accomplished by ‘restitution’ and recovery from activity limitation is accomplished by the combined effect of ‘restitution’ and ‘compensation.’ We aimed to unravel the long-term effects of variation in lesion topography on motor impairment of the hemiparetic lower limb (HLL), and gait capacity as a measure of related activity limitation. Gait was assessed by the 3 m walk test (3MWT) in 67 first-event chronic stroke patients, at their homes. Enduring impairment of the HLL was assessed by the Fugl–Meyer Lower Extremity (FMA-LE) test. The impact of variation in lesion topography on HLL impairment and on walking was analyzed separately for left and right hemispheric damage (LHD, RHD) by voxel-based lesion-symptom mapping (VLSM). In the LHD group, HLL impairment tended to be affected by damage to the posterior limb of the internal capsule (PLIC). Walking capacity tended to be affected by a larger array of structures: PLIC and corona radiata, external capsule and caudate nucleus. In the RHD group, both HLL impairment and walking capacity were sensitive to damage in a much larger number of brain voxels. HLL impairment was affected by damage to the corona radiata, superior longitudinal fasciculus and insula. Walking was affected by damage to the same areas, plus the internal and external capsules, putamen, thalamus and parts of the perisylvian cortex. In both groups, voxel clusters have been found where damage affected FMA-LE and also 3MWT, along with voxels where damage affected only one of the measures (mainly 3MWT). In stroke, enduring ‘activity limitation’ is affected by damage to a much larger array of brain structures and voxels within specific structures, compared to enduring ‘impairment.’ Differences between the effects of left and right hemisphere damage are likely to reflect variation in motor-network organization and post-stroke re-organization related to hemispheric dominance. Further studies with larger sample size are required for the validation of these results.

## Introduction

Stroke is a major disabling condition in the adult population (Sturm et al., 2002; Langhorne et al., 2011). Recovery of voluntary movement in the hemiparetic lower limb (HLL) and regaining walking capacity are primary rehabilitation goals (Vestling and Iwarsson, 2003; Lord et al., 2004). Recovery of walking occurs in 95% of patients within the first 11 weeks after stroke (Jorgensen et al., 1995). However, the pattern of gait often deviates from normality and about one third of the survivors do not get out of home unsupervised (Lord et al., 2004).

Stroke rehabilitation aims to facilitate and enhance the recovery of motor function by a combination of restitution-oriented and compensation-oriented treatment strategies. Restitution-oriented approaches relay on brain plasticity (the capacity of the damaged brain for adaptive re-organization of structure-function relationships), and aim to restore, as much as possible, the characteristics of normal voluntary movement, i.e., strength in discrete muscle groups and especially the normal level of motor control (the capacity to execute the different movements in an isolated and coordinated manner) (Krakauer and Carmichael, 2017). Compensation-oriented approaches are more task oriented. The primary goal is to achieve a better performance in basic and instrumental activities of daily living, with as minimum as possible dependence on others’ help and with maximum safety. With this goal in mind, patients are trained to use their limbs in the most effective way (not necessarily the natural way), without or with external aids, e.g., orthotic devices, cane, etc. (Levin et al., 2009; Krakauer and Carmichael, 2017). In a restitution-oriented strategy, the normal pattern one wishes to restitute is analyzed at the ‘body structures and functions’ level in International Classification of Functioning terminology (World Health Organization, 2002), and deviation from normality is termed ‘impairment.’ In contrast, a compensation-oriented strategy has the ICF ‘activity level’ at its focus, and deviation from normality is termed ‘activity limitation.’ In current stroke-rehabilitation practice both strategies are used. Constraints imposed by treatment costs often dictate a preference for interventions oriented toward achieving independence in basic activities of daily living as quickly as possible. In such cases, restitution-oriented strategies may receive a lesser emphasis (Krakauer and Carmichael, 2017).

Walking-capacity tests, such as the 6-min walk test (6MWT) (Eng et al., 2004) and 3 m walk test (3MWT) (Peters et al., 2014), when conducted in the chronic phase after stroke, reflect the combined effect of restitution and compensation processes on the lasting level of ‘activity limitation,’ as the patient relies on both amelioration of discrete motor functions and use, as necessary, of compensatory measures (orthotics, cane, etc.) in walking. In contrast, the Fugl-Meyer (FM) assessment scale is aimed to determine the level of motor impairment (Gladstone et al., 2002). The FM lower extremity (FMA-LE) reflects mainly the quality of patients’ HLL voluntary movement, thus when it is conducted in the chronic phase, after completion of the recovery process, it represents largely the impact of restitution on the lasting level of ‘impairment.’

Recovery of motor function after a stroke is constrained by lesion topography (Grefkes and Ward, 2014). Lesion studies have focused mainly on the effects of lesion topography on overall motor function (Fries et al., 1993; Miyai et al., 2000; Cheng et al., 2014; Wu et al., 2015) or on the hemiparetic upper limb (HUL) function (Feys et al., 2000; Shelton and Reding, 2001; Wenzelburger et al., 2005; Schiemanck et al., 2008; Lo et al., 2010; Karnath and Rennig, 2017). The effects of lesion characteristics on HLL movement quality and walking capacity were reported in several studies. However, in many of these studies (Miyai et al., 2000; Jayaram et al., 2012; Kaczmarczyk et al., 2012; Chen et al., 2014) lesion location was not determined in a voxel-based manner, producing methodological limitations due to gross parcellation of anatomical templates (e.g., Nadeau et al., 2016), use of a restricted set of regions of interest (e.g., Jayaram et al., 2012), or use of statistical procedures precluding direct inference of structure-function relationships (e.g., Kaczmarczyk et al., 2012).

A few lesion studies avoided some of the above limitations by identifying the anatomical structures essential for HLL movement quality and/or walking capacity using voxel-based lesion symptom mapping (VLSM) analysis (Reynolds et al., 2014; Jones et al., 2016; Moon et al., 2016; Lee et al., 2017; Kim et al., 2018; Handelzalts et al., 2019). In the subacute phase, reduced HLL movement quality (tested by the FMA-LE) was associated with damage to the basal ganglia, insula, internal capsule, and the corona radiata (Moon et al., 2016, 2017). Recently Handelzalts et al. (2019) used VLSM to analyze the effects of left and right hemisphere damage (LHD, RHD) in the subacute phase. In the LHD group, the FMA-LE score was affected by damage to the corticospinal tract in its passage through the corona radiata and the posterior limb of the internal capsule (PLIC), the putamen and the external capsule. In the RHD group, reduced FMA-LE score was associated with lesion to the PLIC only. In another study, by Reynolds et al. (2014), conducted in the chronic phase, reduced FMA-LE score was associated with damage to the corona radiata and the putamen.

Damage to some of the abovementioned structures was found to reduce also walking capacity. The impact of rehabilitation on walking speed in the subacute phase was affected by damage to the putamen, insula, external capsule and neighboring white matter (Jones et al., 2016). In the above study by Handelzalts et al. (2019), the distance subacute LHD stroke patients could cover in 6 min of walking was affected by damage to the same structures that affected the FMA-LE score. RHD patients did not show this relationship. Lee et al. (2017) found the Functional Ambulation Category in the subacute phase to be affected by damage to the internal capsule, lentiform nucleus and cingulum. Yet, Moon et al. (2016) did not find an association between lesion maps and the Functional Ambulation Category in the subacute phase. In the chronic phase, reduced Functional Ambulation Category was associated with damage to the corona radiata, internal capsule, basal ganglia, and primary motor cortex (Lee et al., 2017), and reduced walking speed was associated with damage to the corona radiata and basal ganglia (Reynolds et al., 2014). Kim et al. (2018) found that damage to different brain structures leads to impairment in different gait parameters. For example, damage to the PLIC was associated with reduced walking speed and increased knee extension in the stance phase, and damage to the paracentral lobule was associated with reduced knee flexion in the swing phase and with the reduced ankle dorsiflexion in the stance phase.

The above VLSM studies shed light on the functional neuroanatomy of different gait parameters and HLL motor function (Miyai et al., 2000; Jayaram et al., 2012; Kaczmarczyk et al., 2012; Chen et al., 2014; Nadeau et al., 2016). Damage to the corticospinal tract, basal ganglia and adjacent structures was shown to affect both HLL motor function (impairment level) and walking capacity (activity level). However, these studies did not disclose the extent of network sharing between the two, at the voxel level. As each of the above structures is comprised of hundreds and thousands of voxels, the two levels of analysis could be affected by same or different voxels within a given structure. To determine the extent of voxel sharing between HLL motor function (reflected by the FMA-LE score) and gait capacity (reflected by the 3MWT) we have applied conjunction VLSM analyses. To the best of our knowledge, contrast analyses of lesion effects on movement quality vs. walking capacity, were not done until now in patients with stroke in the chronic phase.

We hypothesized that both HLL function and gait capacity are affected by damage to the cortical and subcortical structures that are directly involved in motor control in the intact brain. However, given the low representation of damage to the lower-limb part of the motor homunculus (anterior cerebral artery territory; ACA) in a cohort comprised typically of middle-cerebral-artery (MCA) strokes, we expected to find “significant” voxels mainly along the corticospinal tract in its passage through the corona radiata and the PLIC (Brodal, 2016). As the level of behavioral performance in the chronic phase reflects not only the impact of damage to the normal functional neuroanatomy of the motor system, but also the neurophysiological factors limiting restitution processes and the capacity to adopt compensatory strategies of motor behavior, we hypothesized that the different sensitivity of FMA-LE and 3MWT to the effects of restitution and compensation will be reflected in a portion of non-shared voxels disclosed by the conjunction VLSM analysis.

## Materials and Methods

### Participants

Sixty-seven first-event stroke patients in the chronic phase (>1 year after onset) who were hospitalized in the subacute period at the Loewenstein Rehabilitation Medical Center, Ra’anana, Israel, were recruited for the study. Patients were included if they did not suffer from previous psychiatric or neurological disorders, their language and cognitive status enabled comprehension of the task requirements, they did not have a subsequent stroke, and they could walk independently or under supervision in the home environment, with or without a walking aid and/or an ankle-foot orthosis. The study was approved by the Ethics Review Board of the Loewenstein Rehabilitation Medical Center (approval number LOE-004-14). The current cohort included subjects who participated also in our recently published study (Frenkel-Toledo et al., 2020), but only those who met the aforementioned inclusion criteria. All participants were informed about the protocol and gave their written informed consent prior to inclusion in the study. Supplementary Table S1 shows the individual demographic and clinical data of all participants.

### Clinical Assessment

The standardized FM (Fugl-Meyer et al., 1975; Gladstone et al., 2002) was used for evaluation of HLL motor ability. The total score of the FMA-LE test is 34. The FM has proven to be reliable and valid in stroke patients (Duncan et al., 1983). The 3MWT was used for evaluation of walking speed (Peters et al., 2014). The 3MWT is a reliable and feasible option when testing of walking has to be done in a limited space, like the home environment. The patients performed three consecutive walking trials at their self-selected walking speed. Participants were allowed 2 m for acceleration/deceleration outside the data collection area, to help reduce gait variability introduced during these phases. Lines were placed on the floor, marking the starting and stopping points for participants, as well as outlining the 3 m timed walking area. The examiner started a stopwatch as soon as the participant’s leg crossed the first marker of the timed walking area and stopped the stopwatch when the participant’s first leg crossed the second marker (Peters et al., 2014). Participants performed the 3MWT wearing their own footwear and they used their mobility device and foot orthosis in case they needed. No manual support was given to the patient during this test.

### Imaging

Follow-up CT scans dated on average 55 and 25 days post stroke onset, for the LHD and RHD groups, respectively. The CTs were carefully examined by a physician experienced in analysis of neuro-imaging data (NS). This was done to ensure that lesion boundaries were clear and traceable, and that the CT presents a stable pattern of tissue damage without a mass effect from residual edema.

### Lesion Analysis

Lesion analyses were performed with the Analysis of Brain Lesions (ABLe) module implemented in MEDx software (Medical-Numerics, Sterling, VA, United States). Lesion delineation was made manually on the digitized CTs. ABLe characterizes brain lesions in MRI and CT scans of the adult human brain by spatially normalizing the lesioned brain into Talairach space using the Montreal Neurological Institute (MNI) template. It reports tissue damage in the normalized brain using an interface to the Talairach Daemon (San Antonio, TX, United States) (Lancaster et al., 2000), Automated Anatomical Labeling (AAL) atlas (Tzourio-Mazoyer et al., 2002; Solomon et al., 2007), Volume Occupancy Talairach Labels (VOTL) atlas (Lancaster et al., 2000; Solomon et al., 2007) or the White Matter Atlas (Mori et al., 2008). Quantification of the amount of tissue damage within each structure/region of the atlas was obtained as described earlier (Haramati et al., 2008). In the current study, tissue damage in the normalized brain was reported using the interface to the AAL and white matter atlases. Registration accuracy of the scans to the MNI template (Woods et al., 1998) was 89.3–95.8% (Mean ± SD: 94.2 ± 1.3%) for the LHD group, and 91.3–95.8% (94.4 ± 0.8%) for the RHD group.

### Voxel-Based Lesion-Symptom Mapping (VLSM)

Voxel-based lesion-symptom mapping (Bates et al., 2003) was used to identify voxels (2 × 2 × 2 mm) of the normalized brain where damage exerts a significant impact on the FMA-LE and 3MWT scores. Voxel-by-voxel analysis was used to calculate the statistical significance of performance difference between subjects with and without damage in a given voxel, using *t*-tests. Only voxels lesioned in at least 15% of subjects were tested, and at least 10 adjacent voxels had to show a statistically significant impact on performance for a cluster of voxels to be reported (McDonald et al., 2017; Frenkel-Toledo et al., 2019). To correct for multiple comparisons, voxels with values exceeding a false discovery rate (FDR)/permutation threshold of *p* < 0.05 were considered significant (Genovese et al., 2002; Mirman et al., 2018). Due to insufficient statistical power in the LHD group, we also report anatomical regions containing clusters of at least 10 voxels, where patients affected in these voxels showed disadvantage relative to patients who were not affected in these voxels, using a lenient criterion of *p* < 0.02, which did not survive FDR correction for multiple comparisons (for a similar approach, see Schoch et al., 2006; Lo et al., 2010; Wu et al., 2015; Moon et al., 2016; Frenkel-Toledo et al., 2019). This information is provided under the assumption that in such cases VLSM results point to trends which are likely to reach significance given a larger study cohort. The maximum *z*-score is reported for each cluster of contiguous above-threshold voxels. Since there may be multiple voxels with this maximum *z*-score in the cluster, we report the coordinates of the voxel that is most superior, posterior and left in its location within the cluster (the centroid of the cluster is not reported as it may not have the highest *z*-score value and it may not be an above-threshold voxel). The Automated Anatomical Labeling atlas (AAL) for gray matter and the White Matter Atlas (Lancaster et al., 2000; Tzourio-Mazoyer et al., 2002; Solomon et al., 2007; Mori et al., 2008) were used to identify the brain structures in which the significant voxel clusters are located. Conjunction analysis was used to classify qualitatively ‘significant’ voxels (corresponding to *z*-scores used for determining results that passed FDR correction for multiple comparisons or, in the case of the LHD group, results that did not survive the FDR correction but passed the more lenient criterion of *z*-score = 2.00) as (1) affecting both FMA-LE and 3MWT; (2) affecting specifically the FMA-LE score (*z* = 2.00 and *z* = 2.98, for LHD and RHD patient groups, respectively); and (3) affecting specifically the 3MWT score (*z* = 2.00 and *z* = 2.38, for LHD and RHD patient groups, respectively).

To rule out the possibility that the results were influenced differently in the LHD and RHD groups by demographic and clinical characteristics, the gender, age, dominance, lesion type, time after stroke onset, lesion volume, FMA-LE score, 3MWT score, FM sensation score, and the proportion of subjects that used an assistive device for walking were compared between groups, using *t*-test, Mann–Whitney test or Chi-square test, as required (normal group distribution of continuous data was assessed using Kolmogorov–Smirnov test). A comparison was made also between the LHD and RHD groups with respect to (1) the proportion of subjects affected in each region of the AAL and WM atlases, and (2) the extent of damage in each region, using Chi-square/Fisher’s exact and Mann-Whitney tests, respectively. To rule out the possibility that the VLSM results were influenced by patient age, follow-up imaging time and lesion volume (Rajashekar et al., 2020), we calculated the correlations between these factors and also the extent of damage in each region of the AAL and WM atlases and the FMA-LE and 3MWT, in the LHD and RHD groups, using Pearson’s correlation or Spearman-rho, as required. FDR was used to correct for multiple correlations between extent of damage in each region of the AAL and WM atlases (68 and 74 regions were damaged in LHD and RHD groups, respectively) and FMA-LE or 3MWT in the LHD and RHD groups. FDR correction was done separately for each behavior measure in each group. Furthermore, the relationship between FMA-LE and 3MWT was evaluated in both groups using Pearson’s correlation. All the tests were done using SPSS (version 25.0) with significance levels of *p*_FDR_ < 0.05.

## Results

The demographic and clinical characteristics of the LHD and RHD patient groups are essentially similar (Table 1). Individual patient data are displayed in Supplementary Table S1.

**TABLE 1:** Demographic and clinical characteristics of participants.

	**LHD group (*n* = 38)**	**RHD group (*n* = 29)**	***p*-Value**
Gender (M/F)	30/8	21/8	0.534^a^
Age: Mean ± SD	60.1 (10.4)	63.7 (10.0)	0.057^c^
Dominance (R/L/A)	36/2/0	25/3/1	0.366^a^
Stroke type (I/H/I > H)	22/16/0	20/8/1	0.273^a^
TAO months: Mean ± SD	28.9 (14.6)	28.7 (10.9)	0.939^b^
Lesion volume (cc): Mean ± SD	25.4 (25.2)	21.5 (28.0)	0.140^c^
FMA-LE: *X*/32: Mean ± SD	26.6 (5.3)	26.1 (7.1)	0.784^b^
3MWT (m/s): Mean ± SD	0.8 (0.3)	0.8 (0.4)	0.970^b^
Sensation (*x*/12): Mean ± SD	9.9 (3.2)	10.3 (2.5)	0.928^c^
*Use of assistive device for walking (no/yes)	26/12	21/8	0.723^a^

LHD, left hemisphere damage; RHD, right hemisphere damage; gender: M, male; F, female; dominance: R, right; L, left, A, ambidextrous; stroke type: I, ischemic; H, hemorrhagic, I > H, ischemic with hemorrhagic transformation; TAO, time after stroke onset; FMA-LE, Fugl-Meyer assessment lower extremity; 3MWT, 3 m walk test; a, Chi-Square test; b, *t*-test; c, Mann–Whitney test. *Assistive device refers in most cases to an ankle-foot orthosis and/or cane. FMA-LE, 3MWT and sensation were tested in the chronic phase (time after stroke onset more than 1 year, as mentioned in “TAO months”).

**FIGURE 1:**
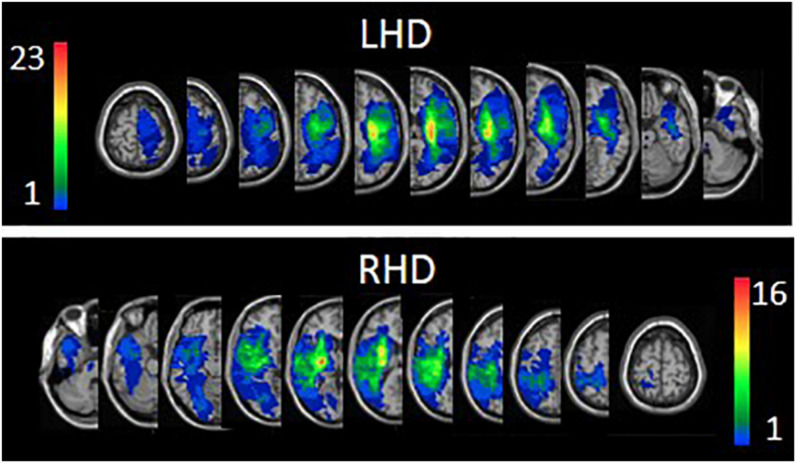
Lesion overlay maps of left hemisphere damage (LHD, *n* = 38) and right hemisphere damage (RHD, *n* = 29) patient groups. Representative normalized slices (out of 90 normalized slices employed) are displayed in radiological convention (right hemisphere on left side and vice versa), with warmer colors indicating greater lesion overlap (units: number of patients with lesion in this region).

The proportion of subjects having damage (yes/no) in each of the regions of the AAL and WM atlases and the average extent of damage in each region were similar in LHD and RHD groups. In both the LHD and RHD groups, age, follow-up imaging time, total hemispheric volume loss and the extent of damage in each atlas region did not correlate with FMA-LE or 3MWT scores. Overlay lesion maps (stroke lesion distribution) of LHD and RHD patients are shown in Figure 1. Individual lesion data are displayed in Supplementary Figure S1. FMA-LE significantly correlated with 3MWT, both in the LHD and RHD groups (*r* = 0.677, *p* < 0.001; *r* = 0.797, *p* < 0.001, respectively).

**FIGURE 2:**
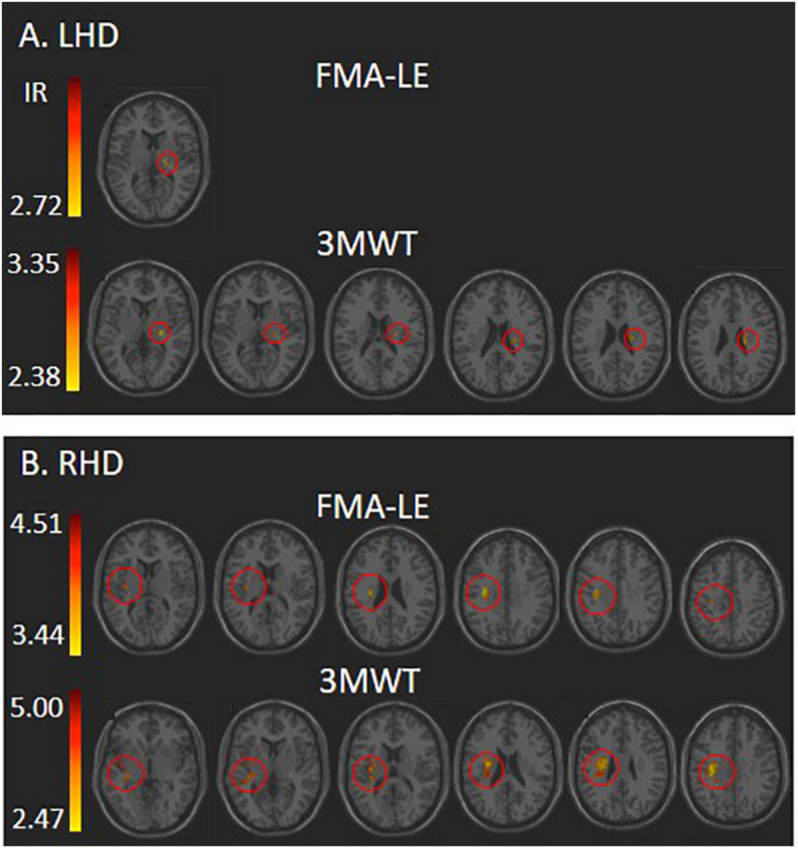
VLSM analysis depicting areas where damage was associated with significantly lower scores of Fugl-Meyer assessment lower extremity (FMA-LE) and 3 m walk test (3MWT) following **(A)** left hemisphere damage (LHD) and **(B)** right hemisphere damage (RHD). Minimum cluster size: 10 voxels, minimum number of patients affected in a voxel (15% of *n*: 6 and 4, respectively). Warmer colors indicate higher *z*-scores. The colored regions in B (RHD) survived FDR correction for multiple comparisons, but the colored regions in **A** (LHD) did not, and represent a lenient criterion of significance – *z*-score ≥ 2.00 (*p* ≤ 0.02). IR, irrelevant, all structures in this case shared a single *z*-score.

Voxel-based lesion-symptom mapping analysis identified voxel clusters in which the existence of damage exerted a significant negative impact on the FMA-LE and 3MWT scores (LHD: Figure 2A and Table 2; RHD: Figure 2B and Table 3). In the LHD group, HLL impairment was affected by damage to brain voxels in the PLIC. Walking capacity was affected by a larger array of structures: PLIC and corona radiata, external capsule and caudate nucleus. These findings rely on a lenient criterion of significance (see Methods section) as they did not survive the FDR correction for multiple comparisons. In the RHD group, both HLL impairment and walking capacity were sensitive to damage in a much larger number of brain voxels. HLL impairment was affected by damage to the corona radiata, superior longitudinal fasciculus and insula. Walking was affected by damage to the same areas, plus the internal and external capsules, putamen, thalamus and parts of the perisylvian cortex. It should be noted that using permutations to correct for multiple comparisons in the VLSM analysis, walking was found to be affected also by damage to the superior corona radiata and superior longitudinal fasciculus. No other results passed the correction for multiple comparisons by permutations.

**TABLE 2:** VLSM results in LHD patients (*n* = 38).

**Test**	**Structure**	***Z*-value**	***X***	***Y***	***Z***	**Voxels**	**% area**
FMA-LE	PLIC	2.72	−18	−14	10	10	2.10
3MWT	SCR	3.35	−26	−16	22	38	4.11
	RLIC	2.71	−26	−18	6	13	4.18
	EC	2.58	−30	−18	6	10	2.22
	Caudate	2.51	−20	−18	24	10	1.04
	PLIC	2.38	−24	−18	10	10	2.10

*FMA-LE, Fugl-Meyer assessment lower extremity; 3MWT, 3 m walk test; VLSM results in this group did not survive the FDR correction for multiple comparisons and are based on a lenient criterion of *z-*score = 2.00 or above (uncorrected *p* < 0.02. The *z-*scores required to pass the correction for multiple comparisons by permutation testing for FMA-LE and 3MWT correspond to 4.26 and 3.77, respectively).*

*EC, external capsule; PLIC/RLIC, posterior/retro-lenticular limb of internal capsule; SCR, superior corona radiata.*

**TABLE 3:** VLSM results in RHD patients (*n* = 29).

**Test**	**Structure**	***Z*-value**	***X***	***Y***	***Z***	**Voxels**	**% area**
FMA-LE	SCR	4.51	30	−16	30	104	11.30
	SLF	4.51	32	−16	30	56	6.79
	Insula	3.82	36	−12	12	16	0.90
	PCR	3.44	30	−22	30	12	2.65
3MWT	SCR^	5.00	30	−14	30	323	35.11
	SLF^	4.67	34	−12	30	283	34.30
	Insula	4.05	32	−14	20	166	9.38
	RLIC	4.06	28	−24	14	133	42.09
	EC	3.67	32	−14	16	92	19.74
	PCR	4.06	30	−22	30	60	13.27
	PLIC	3.52	22	−22	12	56	11.18
	Putamen	3.13	28	−10	14	56	5.26
	Temporal Sup	2.47	44	−6	−6	28	0.89
	Thalamus	3.52	22	−22	12	13	1.23
	Supra Marginal	2.82	50	−42	24	11	0.56

*FMA-LE, Fugl-Meyer lower extremity; 3MWT, 3 m walk test; VLSM results in this group passed FDR correction for multiple comparisons (corresponding in these analyses to *z-*scores of 2.98 for FMA-LE and 2.39 for 3MWT. The *z-*scores required to pass the correction for multiple comparisons by permutation testing for FMA-LE and 3MWT correspond to 4.10 and 3.72, respectively). EC, external capsule; PLIC/RLIC, posterior/retro-lenticular limb of internal capsule; SCR/PCR, superior/posterior corona radiata; SLF, superior longitudinal fasciculus; Temporal Sup, superior temporal gyrus. ^Regions that survived also permutation analysis (SCR: *Z*-value: 5.00; X, Y, Z: 30, −14, 30; Voxels: 72, % of area: 7.83. SLF: *Z*-value: 4.67; X, Y, Z: 34, −12, 30; Voxels: 64, % of area: 7.76).*

**FIGURE 3:**
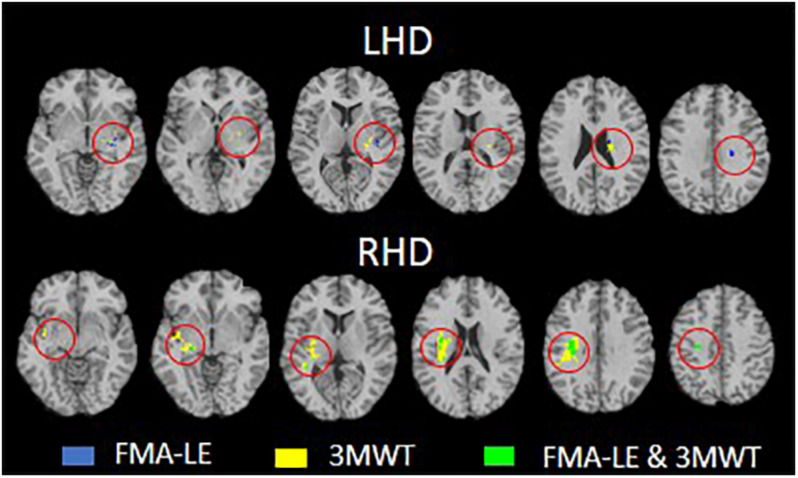
Conjunction analysis depicting areas of brain damage that were associated with lower scores in the Fugl-Meyer assessment lower extremity (FMA-LE) only, 3 m walk test (3MWT) only and both FMA-LE and 3MWT, shown in blue, yellow and green, respectively, in the left hemisphere damage (LHD, *n* = 38) and right hemisphere damage (RHD, *n* = 29) groups.

Voxel-based lesion-symptom mapping conjunction analysis identified voxel clusters in which the existence of damage exerted a significant negative impact on the FMA-LE only, on the 3MWT only, and on both FMA-LE and 3MWT (LHD: Figure 3 upper row and Table 4; RHD: Figure 3 lower row and Table 5). In the LHD group 40% of the voxels where damage affected the FMA-LE were ‘significant’ also for the 3MWT, but only 16% of the voxels where damage affected the 3MWT were ‘significant’ also for the FMA-LE. As can be seen in Table 4, the voxels where damage affected the FMA-LE were located mainly along the corticospinal tract in its passage through the corona radiata and the PLIC, whereas the voxels where damage affected the 3MWT were located in addition to the corticospinal tract also in the putamen and external capsule.

**TABLE 4:** VLSM conjunction analysis in the LHD group.

**Areas**	**FMA-LE only**	**3MWT only**	**FMA-LE plus 3MWT**
SCR	9	36	4
Putamen		19	
PLIC	7	13	9
EC		12	
RLIC		11	4
Heschl gyrus	11		1

*Number of voxels in affected brain regions where damage had a significant impact on FMA-LE only, 3MWT only, and FMA-LE plus 3MWT, in LHD patients (*n* = 38). Only structures with at least 10 voxels affecting performance in one or more of the three options are shown. EC, external capsule; PLIC/RLIC, posterior/retro-lenticular limb of internal capsule; SCR, superior corona radiata.*

**TABLE 5:** VLSM conjunction analysis in the RHD group.

**Areas**	**FMA-LE only**	**3MWT only**	**FMA-LE plus 3MWT**
SLF		227	57
SCR		219	105
RLIC		120	13
EC		83	9
Putamen		54	3
PLIC		49	8
PCR		50	12
Temporal sup		28	
Insula	1	154	19
Thalamus		12	4
Supramarginal		11	

*Number of voxels in affected brain regions where damage had a significant impact on FMA-LE only, 3MWT only, and FMA-LE plus 3MWT, in RHD patients (*n* = 29). Only structures with at least 10 voxels affecting performance in one or more of the three options are shown.*

*EC, external capsule; PLIC/RLIC, posterior/retro-lenticular limb of internal capsule; SCR/PCR, superior/posterior corona radiata; SLF, superior longitudinal fasciculus; temporal sup, superior temporal gyrus.*

In the RHD group (Figure 3 lower row and Table 5) the picture is different. Essentially all the voxels where damage affected the FMA-LE were ‘significant’ also for the 3MWT, while only 19% of the voxels where damage affected the 3MWT were ‘significant’ also for the FMA-LE. As can be seen in Table 5, the voxels where damage affected the FMA-LE were located mainly along the course of the corticospinal tract, like in the LHD group, with the addition of voxels in the superior longitudinal fasciculus, and few voxels also in the external capsule, putamen, insula and the thalamus. As said, practically all these voxels were ‘significant’ also for the 3MWT, yet in all these regions, as well as in parts of the perisylvian cortex, voxels have been found where damage affected the 3MWT in a selective manner.

To ensure that the effects of variation in lesion topography on FMA-LE vs. 3MWT were not influenced by the relatively small sample size of each group, we conducted additional VLSM and conjunction analyses in the entire cohort after flipping the scans onto a single hemisphere template. The results are described in detail in Supplementary Tables S2, S3.

## Discussion

In the current study, we employed conjunction VLSM analysis to unravel the brain voxels critically involved in the formation of enduring impairment vs. enduring activity limitation. Specifically, we checked the impact of variation in lesion topography on HLL motor function (the quality of residual voluntary movement in the HLL, represented here by the FMA-LE score) vs. gait capacity (represented here by the 3MWT score), in the chronic phase. This was done under the assumption that recovery of HLL motor function (impairment level) up to this phase is achieved mainly by a restitution process, whereas recovery of walking capacity (activity level) reflects the combined effect of restitution and compensation processes. We asked whether HLL motor function and gait capacity in the chronic phase, when most neurological recovery is likely to be completed (Ward, 2005; Karnath and Rennig, 2017; Krakauer and Carmichael, 2017) are constrained by damage to the same or to different brain voxels.

Contrary to various earlier VLSM studies that applied lesion flipping onto a single hemisphere template (e.g., Lo et al., 2010; Cheng et al., 2014; Reynolds et al., 2014; Meyer et al., 2016; Lee et al., 2017; Kim et al., 2018), here lesion impact was tested separately for LHD and RHD. This was done to prevent masking of important differences between the hemispheres in structure-function relationships (Frenkel-Toledo et al., 2019; Handelzalts et al., 2019). Indeed, the current study revealed important hemispheric differences. First, the number of ‘significant’ voxels (i.e., voxels in which the existence of damage has a significant negative impact on the studied behavior) was much lower in the LHD group compared to the RHD group. This was true both for the FMA-LE score (indicating the level of enduring motor impairment in the lower limb) and for the 3MWT score (indicating the level of enduring activity limitation, as reflected in walking capacity in the chronic phase). Differences were not restricted to the numbers of ‘significant’ voxels in each hemisphere. Tables 2, 3 show that both FMA-LE and 3MWT were affected by damage to a much smaller range of brain structures following LHD compared to RHD. Finally, contrary to the VLSM results in the RHD group, VLSM analysis following LHD did not survive the FDR correction for multiple comparisons and the results obtained are based on a more lenient criterion, as explained.

The above differences between LHD and RHD do not seem to reflect a selection bias, as the groups did not differ in baseline parameters (gender, age, motor dominance distribution, stroke type, total hemispheric volume loss, extent of damage in the different regions of the AAL and WM atlases, and time after stroke onset). Also, the group average FMA-LE, 3MWT and FM-sensation scores were similar in LHD and RHD patients. The lesion overlay maps (Figure 1) of both LHD and RHD patient groups demonstrate a typical stroke lesion pattern with dominant middle-cerebral artery territory damage (see also Supplementary Figure S1). The existence of marked hemispheric differences in the VLSM results, in spite of the overt similarity between the groups in the above comprehensive set of parameters, points to a possible distinction in structure-function relationships between the hemispheres.

As the large majority of subjects in both groups were right handers (95% and 86% in LHD and RHD groups, respectively), LHD for most subjects reflected damage to the dominant hemisphere and RHD reflected damage to the non-dominant hemisphere. We propose that the above differences in lesion impact between LHD and RHD stem from hemispheric dis-similarity in the functional neuroanatomy of motor control (Tretriluxana et al., 2009; Mani et al., 2013) and in patterns of motor recovery (Zemke et al., 2003; Wu et al., 2015). The processing of sensory-motor data is carried out by a more extensive and densely connected network in the dominant left hemisphere (Guye et al., 2003), thus damage to one network component is more easily substituted by other network components. Left hemisphere dominance for skilled movement is attributed to anatomical and functional hemispheric asymmetries of the primary motor cortex, descending pathways, and somatosensory association and premotor cortices (Serrien et al., 2006). The dominant left hemisphere has a deeper central sulcus (Amunts et al., 1996), a more potent connectivity of intracortical circuits involving the primary motor cortex (Guye et al., 2003; Hammond et al., 2004), a higher excitability of the corticospinal system (De Gennaro et al., 2004), and a higher quality of performance of different motor tasks (Barber et al., 2012).

Lesion studies in the past have already shown that LHD and RHD affect motor functions in a different manner. Chen et al. (2014) reported that RHD is associated with slower and more asymmetric gait in the chronic phase, compared to LHD. Frenkel-Toledo et al. (2019) found a relative paucity of ‘significant’ voxels in LHD compared to RHD, in a VLSM study addressing residual motor function of the hemiparetic upper limb. Handelzalts et al. (2019) found a wider distribution of ‘significant’ voxels for the FMA-LE in LHD compared to RHD. The reasons for the discrepancy between the latter findings (obtained in a smaller cohort) and our current findings are not clear. Possibly, they are related to differences in stroke phase – subacute (around 6 weeks after onset) in Handelzalts et al. (2019) vs. chronic phase (more than 2 years on average) in the current study.

The comparison of the effect of lesion variation on enduring impairment vs. enduring activity limitation yielded interesting results which may indirectly shed new light on the distinction between the neuroanatomical substrate underlying recovery by restitution vs. compensation. By ‘restitution’ we refer in the current study to a natural and treatment-related recovery process which leads to restoration of the characteristics of normal motor activity, i.e., the capacity to recruit enough motor units in a muscle and generate enough force, the capacity to recruit a muscle both as part of a synergy and in isolation, depending on task requirements, the capacity to maintain normal coordination of agonist, antagonist and synergist muscle groups during task performance, and the capacity to benefit from motor learning and exhibit dexterity and acquired skill in task performance (Krakauer and Carmichael, 2017). Restitution following a stroke addresses the ‘impairment’ level of functional analysis, i.e., deviation from normality as expressed at the level of ‘body structure and function’ in WHO-ICF terminology (World Health Organization, 2002). By ‘compensation’ we refer here to accomplishment of a behavioral task using a compensatory bypass mode of operation, e.g., regaining walking capacity using an abnormal gait pattern or using an orthosis. Recovery by compensation has an impact on the ‘activity’ level in WHO-ICF terminology (walking capacity in the current study). It should be noted that our suggested interpretation is based on the conjecture that improvement in HLL motor function (impairment level as reflected in the FMA-LE score), from the onset of stroke up to this chronic stage (in the current cohort, on average about 2.5 years after onset) was achieved mainly by a restitution process, whereas recovery of walking capacity (activity level as reflected in the 3MWT score) was achieved by the combined action of restitution and compensation processes. This conjecture is related to the evidence that most neurological recovery is likely to be completed at this stage (Ward, 2005; Karnath and Rennig, 2017; Krakauer and Carmichael, 2017) and that the FMA-LE unlike the 3MWT is insensitive to compensation thus better reflecting true recovery. However, we do not have data from longitudinal assessments using the FMA-LE and 3MWT measures to support our conclusions concerning the impact of lesion topography on restitution vs. compensation in a direct manner.

The FMA-LE score was used here to assess the impact of lesion topography on HLL enduring motor impairment. Test scores were affected by damage to voxels along the trajectory of the corticospinal tract (PLIC and the corona radiata, in LHD and RHD groups, respectively). In the RHD group, the number of ‘significant’ voxels was much higher and the FMA-LE score was affected also by damage to the superior longitudinal fasciculus and the insula. The 3MWT score was used to assess the impact of lesion topography on enduring activity limitation, as reflected in walking capacity. Test scores were affected not only by damage to the corticospinal tract in different parts of its trajectory, but also by damage to neighboring subcortical and cortical structures – external capsule and basal ganglia in the LHD group (however, based on a lenient criterion, as the results did not survive the FDR correction for multiple comparisons), and the same structures plus a much larger array of brain regions, including the thalamus and parts of the perisylvian cortex, in the RHD group (see Tables 2, 3). Our results are in line with earlier VLSM studies demonstrating that the FMA-LE and walking are affected by lesions involving the corticospinal tract, external capsule, superior longitudinal fasciculus, insula and basal ganglia (Reynolds et al., 2014; Jones et al., 2016; Moon et al., 2016, 2017; Handelzalts et al., 2019; Soulard et al., 2020).

Assessment of the extent of voxel sharing between FMA-LE and 3MWT was conducted using ‘conjunction’ VLSM analyses. These analyses differentiated between voxels in which damage affected specifically either the FMA-LE alone or the 3MWT alone, and voxels where damage affected both the FMA-LE and the 3MWT scores (see Figure 3 and Tables 4, 5). The proportion of voxels that were found significant to 3MWT alone was higher compared to the proportion of voxels that were found significant to the FMA-LE alone or to FMA-LE plus 3WMT. A very small proportion of voxels was found significant to the FMA-LE alone, especially in the RHD group. In both hemispheres, many voxels where damage affected the FMA-LE score were ‘significant’ also for the 3MWT score. In contrast, various brain voxels in different structures where damage affected the 3MWT score were not ‘significant’ for the FMA-LE. These results suggest a role for damage to a larger array of brain structures, and a larger number of voxels within specific structures, in determination of the enduring limitation to walking capacity (activity level) compared to enduring impairment to HLL motor function.

As the analysis was done in the chronic phase, when further amelioration of function is unlikely to occur (Ward, 2005; Karnath and Rennig, 2017; Krakauer and Carmichael, 2017), the impact of lesion topography on the deviation from normality as reflected in the FMA-LE score (impairment level) points to a role for the ‘significant’ voxels (largely along the corticospinal tract) in mediation of motor control in the intact brain. However, part of these voxels (probably those in the less ‘traditional’ regions of motor functional neuroanatomy), are assumed to play a critical role in adaptive re-mapping and re-organization processes of ‘restitution’ in the recovery post stroke. In contrast, lesion impact on the 3MWT score (activity level), points to a role for the ‘significant’ voxels (corticospinal tract and other regions) in mediation of gait and its recovery by the combined effect of restitution and compensation.

### Limitations

Several caveats of the current study need to be taken into consideration. First, stroke may affect walking capacity in different ways and not only through impairment to HLL voluntary movement captured by the FMA-LE (e.g., by damage to neurophysiological mechanisms underlying postural reactions). Thus, the ‘impairment’ level of analysis from which emerged the ‘activity limitation’ as reflected in the 3MWT score could be captured only in part in the current study. This means that the surplus of voxels ‘significant’ only for the 3MWT (and not for the FMA-LE), points not only to voxels recruited by ‘compensation’ in the process of recovery. It may as well point to structural elements contributing to other components of the ‘impairment’ level, not captured by the FMA-LE. Second, the inference made from the assessment of long-term ‘impairment’ vs. ‘activity-limitation’ on ‘restitution’ vs. ‘compensation’ processes of recovery, is necessarily indirect and partial, as the FMA-LE score, and not only the 3MWT score, contains parts that are sensitive to amelioration by ‘compensation’ or simply by training-related increment in muscle strength. Third, the large majority of patients in the current cohort had strokes located within the MCA territory. This limited the possibility to identify ‘significant’ voxel clusters in other vascular territories, including the anterior cerebral artery (ACA) territory which supplies parts of the motor cortex where the cell bodies of pyramidal neurons controlling lower-limb movement reside, and posterior-circulation strokes affecting the corticospinal tract in its passage through the ventral part of the brainstem. Fourth, the results in the LHD group are based on a lenient criterion, as they did not survive the FDR correction for multiple comparisons. This fact could contribute to the hemispheric differences in VLSM results. Future studies with larger cohorts, enabling application of comparable correction methods, are expected to clarify this matter, while the current LHD results might be better considered as possibly being contaminated by type-1 error. Correction for multiple comparisons by permutation should be used in future studies with the larger samples because FDR, although used in various past VLSM studies, is currently considered too liberal (Mirman et al., 2018). Fifth, the generalizability of the results may be partial, because the studied cohort included only stroke patients in the chronic stage (on average, about 2.5 years after the onset of stroke) who were hospitalized in the subacute period at the Loewenstein Rehabilitation Medical Center, where admission is biased toward a population somewhat younger than the average stroke population. We also set neurological or psychiatric co-morbidity as an exclusion criterion in order to limit the possible impact of additional factors. We recruited only subjects in whom stroke-related language and cognitive problems did not preclude clear understanding of task requirements, thus further limiting generalizability to the entire stroke population. This limitation is probably balanced by the benefits of clearer segregation, as the marked heterogeneity and multiplicity of co-variates within the general stroke population tends to blur important relationships between lesion data and functional outcomes that can be shown in segregated subgroups. Sixth, data collection was conducted in the chronic stage, mostly in the patients’ homes. Possible impact of residual chronic aphasia among LHD patients and residual spatial neglect among RHD patients (Katz et al., 1999; Ginex et al., 2020) could not be assessed on the basis of formal testing at that time (however, patients were excluded if the examiner had the impression that either language or cognitive problems precluded clear understanding of the requirements of the studied tasks). Lastly, despite the fact that in both the LHD and RHD groups, age, follow-up imaging time, total hemispheric volume loss and the extent of damage in each atlas region did not correlate with either the FMA-LE score or the 3MWT score, it is still possible that VLSM studies analyzing these variables as covariates (such analysis cannot be conducted in the MEDx software we use) will refine the results, as suggested recently by Rajashekar et al. (2020).

## Conclusion

In stroke, enduring ‘activity limitation’ is affected by damage to a much larger array of brain structures and voxels within specific structures, compared to enduring ‘impairment.’ Differences between the effects of left and right hemisphere damage are likely to reflect variation in motor-network organization and post-stroke re-organization related to hemispheric dominance. However, as the findings of the LHD group rely on a lenient criterion (as they did not survive the FDR correction for multiple comparisons), further studies are required for validation of trends pointed by the current results.

## Data Availability Statement

The raw data supporting the conclusions of this article will be made available by the authors, without undue reservation.

## Ethics Statement

The studies involving human participants were reviewed and approved by Ethics Review Board of the Loewenstein Rehabilitation Medical Center. The patients/participants provided their written informed consent to participate in this study.

## Author Contributions

SF-T was involved in planning and conducting the experiments as well as data analysis, interpretation, and drafting of the manuscript. SO-G was involved in conducting the experiments as well as data analysis, interpretation of data, and revising of the manuscript. LM was involved in conducting the experiments as well as data analysis. OG was involved in conducting the experiments as well as data analysis. NS was involved in subject medical screening, lesion delineation, planning the experiment, interpretation, and revising of the manuscript. All authors read and approved the final manuscript.

## Conflict of Interest

The authors declare that the research was conducted in the absence of any commercial or financial relationships that could be construed as a potential conflict of interest.

## References

[B1] Amunts K. Schlaug G. Schleicher A. Steinmetz H. Dabringhaus A. Roland P. E. (1996). Asymmetry in the human motor cortex and handedness. *Neuroimage* 4 216–222. 10.1152/jn.1998.79.4.2149 9345512

[B2] Barber A. D. Srinivasan P. Joel S. E. Caffo B. S. Pekar J. J. Mostofsky S. H. (2012). Motor “dexterity”: evidence that left hemisphere lateralization of motor circuit connectivity is associated with better motor performance in children. *Cereb. Cortex* 22 51–59. 10.1093/cercor/bhr062 21613469 PMC3236793

[B3] Bates E. Wilson S. M. Saygin A. P. Dick F. Sereno M. I. Knight R. T. (2003). Voxel-based lesion-symptom mapping. *Nat. Neurosci.* 6 448–450. 10.1038/nn1050 12704393

[B4] Brodal P. (2016). *The Central Nervous System.* 5th Edn New York, NY: Oxford UP.

[B5] Chen I. H. Novak V. Manor B. (2014). Infarct hemisphere and noninfarcted brain volumes affect locomotor performance following stroke. *Neurology* 82 828–834. 10.1212/WNL.0000000000000186 24489132 PMC3959753

[B6] Cheng B. Forkert N. D. Zavaglia M. Hilgetag C. C. Golsari A. Siemonsen S. (2014). Influence of stroke infarct location on functional outcome measured by the modified rankin scale. *Stroke* 45 1695–1702. 10.1161/STROKEAHA.114.005152 24781084

[B7] De Gennaro L. Cristiani R. Bertini M. Curcio G. Ferrara M. Fratello F. (2004). Handedness is mainly associated with an asymmetry of corticospinal excitability and not of transcallosal inhibition. *Clin. Neurophysiol.* 115 1305–1312. 10.1016/j.clinph.2004.01.014 15134697

[B8] Duncan P. W. Propst M. Nelson S. G. (1983). Reliability of the Fugl-Meyer assessment of sensorimotor recovery following cerebrovascular accident. *Phys. Ther.* 63 1606–1610. 10.1093/ptj/63.10.1606 6622535

[B9] Eng J. J. Dawson A. S. Chu K. S. (2004). Submaximal exercise in persons with stroke: test-retest reliability and concurrent validity with maximal oxygen consumption. *Arch. Phys. Med. Rehabil.* 85 113–118. 10.1016/S0003-9993(03)00436-2 14970978 PMC3167868

[B10] Feys H. Hetebrij J. Wilms G. Dom R. De Weerdt W. (2000). Predicting arm recovery following stroke: value of site of lesion. *Acta Neurol. Scand.* 102 371–377. 10.1034/j.1600-0404.2000.102006371.x 11125752

[B11] Frenkel-Toledo S. Fridberg G. Ofir S. Bartur G. Lowenthal-Raz J. Granot O. (2019). Lesion location impact on functional recovery of the hemiparetic upper limb. *PLoS One* 14:e0219738. 10.1371/journal.pone.0219738 31323056 PMC6641167

[B12] Frenkel-Toledo S. Ofir-Geva S. Soroker N. (2020). Lesion topography impact on shoulder abduction and finger extension following left and right hemispheric stroke. *Front. Human Neurosci.* 14:282. 10.3389/fnhum.2020.00282 32765245 PMC7379861

[B13] Fries W. Danek A. Scheidtmann K. Hamburger C. (1993). Motor recovery following capsular stroke. Role of descending pathways from multiple motor areas. *Brain* 116 369–382. 10.1093/brain/116.2.369 8461971

[B14] Fugl-Meyer A. R. Jääskö L. Leyman I. Olsson S. Steglind S. (1975). The post-stroke hemiplegic patient. 1. a method for evaluation of physical performance. *Scand. J. Rehabil. Med.* 7 13–31. 1135616

[B15] Genovese C.R. Lazar N.A. Nichols T. (2002). Thresholding of statistical maps in functional neuroimaging using the false discovery rate. *Neuroimage* 15 870–878. 10.1006/nimg.2001.1037 11906227

[B16] Ginex V. Gilardone G. Viganò M. Monti A. Judica E. Passaro I. (2020). Interaction between recovery of motor and language abilities after stroke. *Arch. Phys. Med. Rehabil.* 101 1367–1376. 10.1016/j.apmr.2020.04.010 32417441

[B17] Gladstone D. J. Danells C. J. Black S. E. (2002). The fugl-meyer assessment of motor recovery after stroke: a critical review of its measurement properties. *Neurorehabil. Neural Repair* 16 232–240. 10.1177/154596802401105171 12234086

[B18] Grefkes C. Ward N.S. (2014). Cortical reorganization after stroke: how much and how functional? *Neuroscientist* 20 56–70. 10.1177/1073858413491147 23774218

[B19] Guye M. Parker G. J. M. Symms M. Boulby P. Wheeler-Kingshott C. A. M. Salek-Haddadi A. (2003). Combined functional MRI and tractography to demonstrate the connectivity of the human primary motor cortex in vivo. *Neuroimage* 19 1349–1360. 10.1016/S1053-8119(03)00165-4 12948693

[B20] Hammond G. Faulkner D. Byrnes M. Mastaglia F. Thickbroom G. (2004). Transcranial magnetic stimulation reveals asymmetrical efficacy of intracortical circuits in primary motor cortex. *Exp. Brain Res.* 155 19–23. 10.1007/s00221-003-1696-x 15064880

[B21] Handelzalts S. Melzer I. Soroker N. (2019). Analysis of brain lesion impact on balance and gait following stroke. *Front. Hum. Neurosci.* 13:149. 10.3389/fnhum.2019.00149 31139067 PMC6527742

[B22] Haramati S. Soroker N. Dudai Y. Levy D. A. (2008). The posterior parietal cortex in recognition memory: a neuropsychological study. *Neuropsychologia* 46 1756–1766. 10.1016/j.neuropsychologia.2007.11.015 18178228

[B23] Jayaram G. Stagg C. J. Esser P. Kischka U. Stinear J. Johansen-berg H. (2012). Clinical neurophysiology relationships between functional and structural corticospinal tract integrity and walking post stroke. *Clin. Neurophysiol.* 123 2422–2428. 10.1016/j.clinph.2012.04.026 22717679 PMC3778984

[B24] Jones P. S. Pomeroy V. M. Wang J. Schlaug G. Marrapu S. T. Geva S. (2016). Does stroke location predict walk speed response to gait rehabilitation? *Hum. Brain Mapp.* 37 689–703. 10.1002/hbm.23059 26621010 PMC4738376

[B25] Jorgensen H. S. Nakayama H. Raaschou H. O. Olsen T. S. (1995). Recovery of walking function in stroke patients: the Copenhagen Stroke Study. *Arch. Phys. Med. Rehabil.* 76 27–32. 10.1016/s0003-9993(95)80038-7 7811170

[B26] Kaczmarczyk K. Wit A. Krawczyk M. Zaborski J. Gajewski J. (2012). Associations between gait patterns, brain lesion factors and functional recovery in stroke patients. *Gait Posture* 35 214–217. 10.1016/j.gaitpost.2011.09.009 21937234

[B27] Karnath H. Rennig J. (2017). Investigating structure and function in the healthy human brain: validity of acute versus chronic lesion-symptom mapping. *Brain Struct. Funct.* 222 2059–2070. 10.1007/s00429-016-1325-7 27807627

[B28] Katz N. Hartman-Maeir A. Ring H. Soroker N. (1999). Functional disability and rehabilitation outcome in right hemisphere damaged patients with and without unilateral spatial neglect. *Arch. Phys. Med. Rehabil.* 80 379–384. 10.1016/s0003-9993(99)90273-3 10206598

[B29] Kim D. H. Kyeong S. Do K. H. Lim S. K. Cho H. K. Jung S. (2018). Brain mapping for long-term recovery of gait after supratentorial stroke: a retrospective cross-sectional study. *Medicine (Baltimore)* 97:e0453. 10.1097/MD.0000000000010453 29668613 PMC5916674

[B30] Krakauer J. W. Carmichael S. T. (2017). *Broken Movement: The Neurobiology of Motor Recovery After Stroke.* Cambridge, MA: MIT Press

[B31] Lancaster J. L. Woldorff M. G. Parsons L. M. Liotti M. Freitas C. S. Rainey L. (2000). Automated talairach atlas labels for functional brain mapping. *Hum. Brain Mapp.* 10 120–131. 10.1002/1097-0193(200007)10:3<120::aid-hbm30>3.0.co;2-8 10912591 PMC6871915

[B32] Langhorne P. Bernhardt J. Kwakkel K. (2011). Stroke rehabilitaiton. *Lancet* 377 1693–1702. 10.1016/S0140-6736(11)60325-5 21571152

[B33] Lee K. B. Kim J. S. Hong B. Y. Sul B. Song S. Sung W. J. (2017). Brain lesions affecting gait recovery in stroke patient. *Brain Behav.* 7:e00868. 10.1002/brb3.868 29201557 PMC5698874

[B34] Levin M. F. Kleim J. A. Wolf S. L. (2009). What do motor “recovery” and “compensation” mean in patients following stroke? *Neurorehabil. Neural Repair.* 23(4), 313–319. 10.1177/1545968308328727 19118128

[B35] Lo R. Gitelman D. Levy R. Hulvershorn J. Parrish T. (2010). Identification of critical areas for motor function recovery in chronic stroke subjects using voxel-based lesion symptom mapping. *Neuroimage* 49 9–18. 10.1016/j.neuroimage.2009.08.044 19716427

[B36] Lord S. E. Mcpherson K. Mcnaughton H. K. Rochester L. Weatherall M. (2004). Community ambulation after atroke: how important and obtainable is it and what measures appear predictive? *Arch. Phys. Med. Rehabil.* 85 234–239. 10.1016/j.apmr.2003.05.002 14966707

[B37] Mani S. Mutha P. K. Przybyla A. Haaland K. Y. Good D. C. Sainburg R. L. (2013). Contralesional motor deficits after unilateral stroke reflect hemisphere-specific control mechanisms. *Brain* 136 1288–1303. 10.1093/brain/aws283 23358602 PMC3613707

[B38] McDonald V. Hauner K. K. Chau A. Krueger F. Grafman J. (2017). Networks underlying trait impulsivity: evidence from voxel-based lesion-symptom mapping. *Hum. Brain Mapp.* 38 656–665. 10.1002/hbm.23406 27667777 PMC5225118

[B39] Meyer S. Kessner S. S. Cheng B. Bönstrup M. Schulz R. Hummel F. C. (2016). Voxel-based lesion-symptom mapping of stroke lesions underlying somatosensory deficits. *Neuroimage Clin.* 10 257–266. 10.1016/j.nicl.2015.12.005 26900565 PMC4724038

[B40] Mirman D. Landrigan J. F. Kokolis S. Verillo S. Ferrara C. Pustina D. (2018). Corrections for multiple comparisons in voxel-based lesion-symptom mapping. *Neuropsychologia* 115 112–123. 10.1016/j.neuropsychologia.2017.08.025 28847712 PMC5826816

[B41] Miyai I. Suzuki T. Kang J. Volpe B. T. (2000). Improved functional outcome in patients with hemorrhagic stroke in putamen and thalamus compared with those with stroke restricted to the putamen or thalamus. *Stroke* 31 1365–1369. 10.1161/01.str.31.6.1365 10835458

[B42] Moon H. I. Lee H. J. Yoon S. Y. (2017). Lesion location associated with balance recovery and gait velocity change after rehabilitation in stroke patients. *Neuroradiology* 59 609–618. 10.1007/s00234-017-1840-0 28523357

[B43] Moon H. I. Pyun S. B. Tae W. S. Kwon H. K. (2016). Neural substrates of lower extremity motor, balance, and gait function after suprat entorial stroke using voxel-based lesion symptom mapping. *Neuroradiology* 58 723–731. 10.1007/s00234-016-1672-3 26961307

[B44] Mori S. Oishi K. Jiang H. Jiang L. Li X. Akhter K. (2008). Stereotaxic white matter atlas based on diffusion tensor imaging in an ICBM templat. *Neuroimage* 40 570–582. 10.1016/j.neuroimage.2007.12.035 18255316 PMC2478641

[B45] Nadeau S. E. Dobkin B. Wu S. S. Pei Q. Duncan P. W. LEPAS Investigative team (2016). The effects of stroke type, locus, and extent on long-term outcome of gait rehabilitation: the LEAPS experience. *Neurorehabil. Neural Repair* 30 615–625. 10.1177/1545968315613851 26498434 PMC4842161

[B46] Peters D. M. Middleton A. Donley J. W. Blanck E. L. Fritz S. L. (2014). Concurrent validity of walking speed values calculated via the GAITRite electronic walkway and 3 meter walk test in the chronic stroke population. *Physiother. Theory Pract.* 30 183–188. 10.3109/09593985.2013.845805 24164441 PMC4251769

[B47] Rajashekar D. Wilms M. Hecker K. G. Hill M. D. Dukelow S. Fiehler J. (2020). The impact of covariates in voxel-wise lesion-symptom mapping. *Front. Neurol.* 11:854. 10.3389/fneur.2020.00854 32922356 PMC7456820

[B48] Reynolds A. M. Peters D. M. Vendemia J. M. C. Smith L. P. Sweet R. C. Baylis G. C. (2014). Neuronal injury in the motor cortex after chronic stroke and lower limb motor impairment: a voxel- based lesion symptom mapping study. *Neural Regen. Res.* 9 766–772. 10.4103/1673-5374.131589 25206888 PMC4146271

[B49] Schiemanck S. K. Kwakkel G. Post M. W. M. Kappelle L. J. Prevo A. J. H. (2008). Original report impact of internal capsule lesions on outcome of motor hand function at one year post-stroke. *J. Rehabil. Med.* 40 96–101. 10.2340/16501977-0130 18509572

[B50] Schoch B. Dimitrova A. Gizewski E. R. Timmann D. (2006). Functional localization in the human cerebellum based on voxelwise statistical analysis: a study of 90 patients. *Neuroimage* 30 36–51. 10.1016/j.neuroimage.2005.09.018 16253526

[B51] Serrien D. J. Ivry R. B. Swinnen S. P. (2006). Dynamics of hemispheric specialization and integration in the context of motor control. *Nat. Rev. Neurosci.* 7 160–166. 10.1038/nrn1849 16429125

[B52] Shelton F. N. Reding M. J. (2001). Effect of lesion location on upper limb motor recovery after stroke. *Stroke* 32 107–112. 10.1161/01.str.32.1.107 11136923

[B53] Solomon J. Raymont V. Braun A. Butman J. A. Grafman J. (2007). User-friendly software for the analysis of brain lesions (ABLe). *Comput. Methods Programs Biomed.* 86 245–254. 10.1016/j.cmpb.2007.02.006 17408802 PMC1995425

[B54] Soulard J. Huber C. Baillieul S. Thuriot A. Renard F. Broche B. A. (2020). Motor tract integrity predicts walking recovery: a diffusion MRI study in subacute stroke. *Neurology* 94 e583–e593. 10.1212/WNL.0000000000008755 31896618

[B55] Sturm J. W. Dewey H. M. Donnan G. A. Macdonell R. A. McNeil J. J. Thrift A. G. (2002). Handicap after stroke: how does it relate to disability, perception of recovery, and stroke subtype? The North East Melbourne Stroke Incidence Study (NEMESIS). *Stroke* 33 762–768. 10.1161/hs0302.103815 11872901

[B56] Tretriluxana J. Gordon J. Fisher B. E. Winstein C. J. (2009). Hemisphere specific impairments in reach-to-grasp control after stroke: effects of object size. *Neurorehabil. Neural Repair* 23. 679–691. 10.1177/1545968309332733 19411406

[B57] Tzourio-Mazoyer N. Landeau B. Papathanassiou D. Crivello F. Etard O. Delcroix N. (2002). Automated anatomical labeling of activations in SPM Using a macroscopic anatomical parcellation of the MNI MRI single-subject brain. *Neuroimage* 15 273–289. 10.1006/nimg.2001.0978 11771995

[B58] Vestling M. Iwarsson S. (2003). Indicators for return to work after stroke and the importance of work for subjective well-being and life satisfaction. *J. Rehabil. Med.* 35 127–131. 10.1080/16501970310010475 12809195

[B59] Ward N. S. (2005). Mechanisms underlying recovery of motor function after stroke, Postgrad. *Med. J.* 81 510–514. 10.1136/pgmj.2004.030809 16085742 PMC1743338

[B60] Wenzelburger R. Kopper F. Frenzel A. Stolze H. Klebe S. Brossmann A. (2005). Hand coordination following capsular stroke. *Brain* 128 64–74. 10.1093/brain/awh317 15471902

[B61] Woods R. P. Grafton S. T. Watson J. D. Sicotte N. L. Mazziotta J. C. (1998). Automated image registration: II. Intersubject validation of linear and nonlinear models. *J. Comput. Assist. Tomogr.* 22 153–165. 10.1097/00004728-199801000-00028 9448780

[B62] World Health Organization (2002). *Towards a Common Language for Functioning, Disability and Health: ICF.* Geneva, World Health Organization.

[B63] Wu O. Cloonan L. Mocking S. J. T. Bouts M. J. R. J. Copen W. A. Cougo-Pinto P. T. (2015). Role of acute lesion topography in initial ischemic stroke severity and long-term functional outcomes. *Stroke* 46 2438–2444. 10.1161/STROKEAHA.115.009643 26199314 PMC4550548

[B64] Zemke A. C. Heagerty P. J. Lee C. Cramer S. C. (2003). Motor cortex organization after stroke is related to side of stroke and level of recovery. *Stroke* 34 e23–e28. 10.1161/01.STR.0000065827.35634.5E 12677024

