# Supplementary figures and images for: Stroke Lesion Impact on Lower Limb Function
Source: Front Hum Neurosci. 2021 Feb 1;15:592975. doi: 10.3389/fnhum.2021.592975 (PMC7882502; doi:10.3389/fnhum.2021.592975)

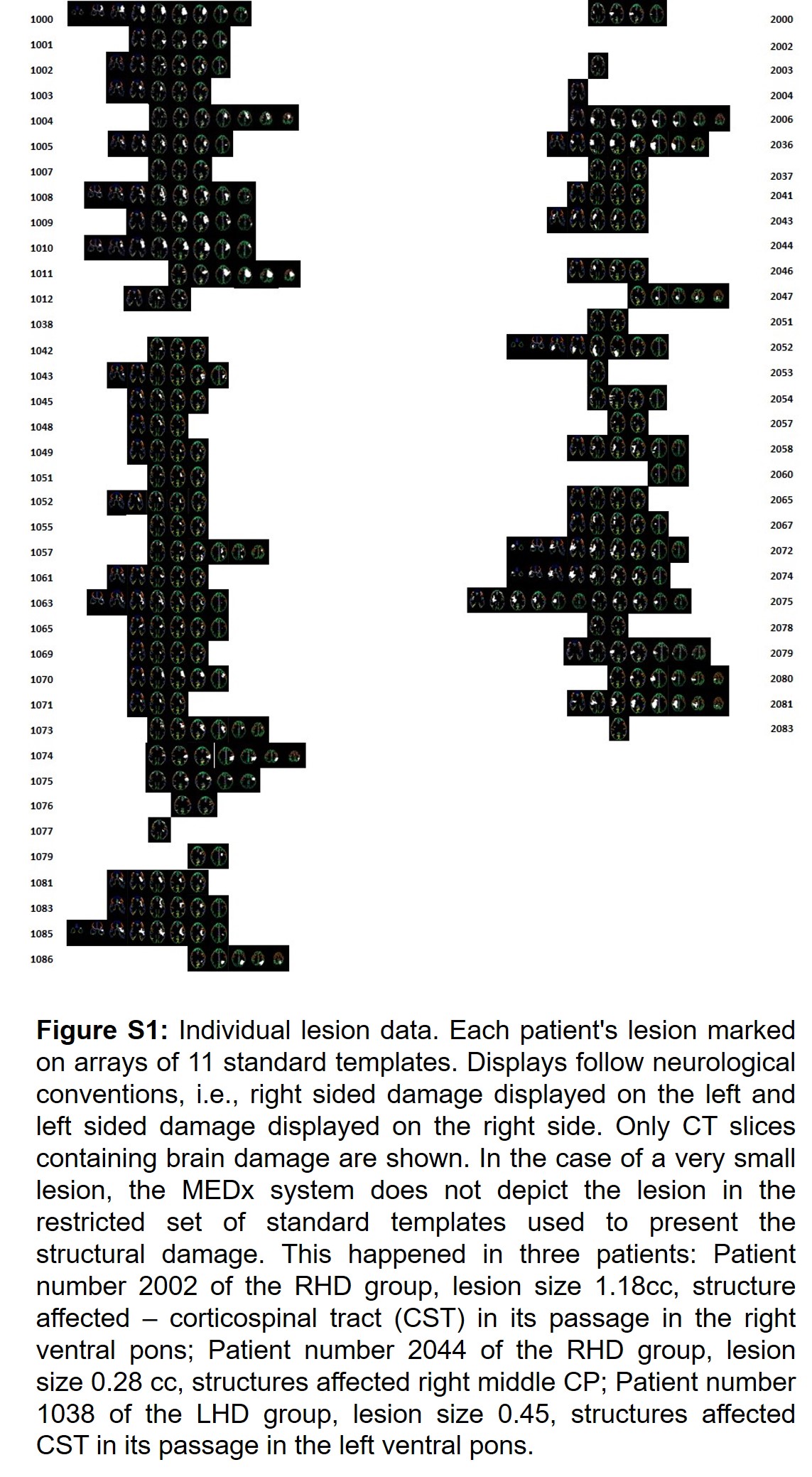

Supplement: Supplementary file 1 [file Image_1.JPEG]
