# Supplementary material for: Stroke Lesion Impact on Lower Limb Function
Source: Front Hum Neurosci. 2021 Feb 1;15:592975. doi: 10.3389/fnhum.2021.592975 (PMC7882502; doi:10.3389/fnhum.2021.592975)
Supplement: Supplementary file 3 [file Table_2.DOCX]

**Table S2:**

Title: VLSM analysis of the entire cohort (LHD and RHD together, n = 67).

| **Test** | **Structure** | **Z-value** | **X** | **Y** | **Z** | **Voxels** | **% area** |
| --- | --- | --- | --- | --- | --- | --- | --- |
| **FMA-LE** | SCR | 4.15 | -30 | -16 | 30 | 194 | 21.00 |
|  | Insula | 3.41 | -40 | -10 | 4 | 183 | 9.85 |
|  | SLF | 3.99 | -32 | -16 | 30 | 106 | 13.01 |
|  | RLIC | 3.27 | -32 | -26 | 4 | 67 | 21.54 |
|  | EC | 3.37 | -32 | -10 | 6 | 67 | 14.89 |
|  | PLIC | 3.75 | -18 | -14 | 10 | 44 | 9.22 |
|  | Heschl | 3.57 | -36 | -24 | 14 | 56 | 24.89 |
|  | Putamen | 2.99 | -30 | -10 | 6 | 38 | 3.77 |
|  | PCR | 2.74 | -28 | -24 | 32 | 19 | 4.26 |
|  | Temporal Sup | 2.60 | -40 | -4 | -8 | 19 | 0.83 |
|  | IFO | 2.99 | -32 | -18 | -4 | 17 | 2.02 |
|  | Thalamus | 3.75 | -18 | -14 | 10 | 10 | 0.91 |
|  | R. operculum | 2.81 | -36 | -28 | 20 | 10 | 1.01 |
|  | Supramarginal | 2.14 | -46 | -44 | 26 | 10 | 0.80 |
| **3MWT** | SCR | 4.23 | -26 | -16 | 24 | 183 | 19.81 |
|  | SLF | 3.81 | -32 | -16 | 30 | 86 | 10.55 |
|  | RLIC | 3.61 | -32 | -26 | 4 | 62 | 19.94 |
|  | EC | 3.44 | -28 | -12 | 18 | 61 | 13.56 |
|  | PLIC | 3.41 | -26 | -18 | 14 | 49 | 10.27 |
|  | Putamen | 3.43 | -26 | -8 | 10 | 22 | 2.18 |
|  | Insula | 3.20 | -38 | -2 | 6 | 20 | 1.08 |
|  |  | 3.23 | -32 | -12 | 20 | 17 | 0.91 |
|  | PCR | 3.29 | -24 | -22 | 26 | 18 | 4.04 |
|  | Heschl gyrus | 3.41 | -34 | -22 | 4 | 17 | 7.56 |
| **3MWT*** | SCR | 4.23 | -26 | -16 | -24 | 313 | 33.87 |
|  | SLF | 3.81 | -32 | -16 | 30 | 193 | 23.68 |
|  | PLIC | 3.41 | -26 | -18 | 14 | 155 | 32.49 |
|  | EC | 3.44 | -28 | -12 | 18 | 132 | 29.33 |
|  | Insula | 3.23 | -32 | -12 | 20 | 232 | 12.49 |
|  | RLIC | 3.61 | -32 | -26 | 4 | 110 | 35.37 |
|  | Putamen | 3.43 | -26 | -8 | -10 | 108 | 10.70 |
|  | Heschl | 3.41 | -34 | -22 | 4 | 54 | 24.00 |
|  | PCR | 3.29 | -24 | -22 | 26 | 48 | 10.76 |
|  | Thalamus | 3.33 | -22 | -18 | 2 | 33 | 3.00 |
|  | IFO | 3.30 | -32 | -18 | -4 | 21 | 8.68 |
|  | Supramarginal | 2.85 | -46 | -40 | 26 | 17 | 1.35 |

VLSM results of FMA-LE did not survive the FDR correction for multiple comparisons and are based on a lenient criterion (z score = 2.00 or above; uncorrected p < 0.02). VLSM of 3MWT passed FDR correction for multiple comparisons (corresponding in these analyses to z scores of 2.58). The z scores required to pass the correction for multiple comparisons by permutation testing for FMA-LE and 3MWT correspond to 4.18 and 3.80, respectively. *VLSM results of 3MWT that are based on a lenient criterion (z score = 2.00; uncorrected p < 0.02) are presented as well.

FMA-LE = Fugl-Meyer assessment lower extremity; 3MWT = 3-meter walk test; EC = external capsule; LHD/RHD = left/right hemisphere damage; PLIC/RLIC = posterior/retro-lenticular limb of internal capsule; IFO = inferior fronto-occipital fasciculus; PCR/SCR = posterior/superior corona radiata; R. operculum = Rolandic operculum; SLF = superior longitudinal fasciculus; Temporal Sup = temporal superior gyrus.
