# Supplementary material for: Stroke Lesion Impact on Lower Limb Function
Source: Front Hum Neurosci. 2021 Feb 1;15:592975. doi: 10.3389/fnhum.2021.592975 (PMC7882502; doi:10.3389/fnhum.2021.592975)
Supplement: Supplementary file 4 [file Table_3.DOCX]

**Table S3:**

Title: VLSM conjunction analysis in the entire cohort (LHD and RHD together, n =67), using a lenient criterion for both FMA-LE and 3MWT.

| **Areas** | **FMA-LE only** | **3MWT only** | **FMA-LE plus 3MWT** |
| --- | --- | --- | --- |
| Insula | 32 | 82 | 152 |
| Heschl | 15 | 13 | 41 |
| Temp Sup | 15 | 3 | 8 |
| Putamen | 4 | 74 | 34 |
| Thalamus | 1 | 19 | 14 |
| Supramrginal | 0 | 8 | 10 |
| PLIC | 4 | 106 | 49 |
| RLIC | 1 | 43 | 67 |
| SCR | 9 | 126 | 187 |
| PCR | 0 | 29 | 19 |
| EC | 1 | 66 | 66 |
| SLF | 4 | 89 | 104 |
| IFO | 1 | 5 | 16 |

Number of voxels in affected brain regions where damage had a significant impact on FMA-LE only (z = 2), 3MWT only (z = 2), and FMA-LE plus 3MWT, in LHD and RHD patients (n = 67). Only structures with at least 10 voxels affecting performance in one or more of the 3 options are shown. EC = external capsule; IFO = inferior fronto-occipital fasciculus; LHD/RHD = left/right hemisphere damage; PLIC/RLIC = posterior/retro-lenticular limb of internal capsule; SCR/PCR = superior/posterior corona radiata; SLF = superior longitudinal fasciculus; Temporal Sup = superior temporal gyrus.
